# Identification of *MsCYP79* and *MsCYP83* gene families and its response to mechanical damage in *Medicago sativa* L.

**DOI:** 10.1371/journal.pone.0322981

**Published:** 2025-05-08

**Authors:** Fang Wu, Jing Zhang, Hongshan Yang, Huirong Duan

**Affiliations:** Lanzhou Institute of Husbandry and Pharmaceutical Sciences, Chinese Academy of Agricultural Sciences, Lanzhou, Gansu, China; North Dakota State University, UNITED STATES OF AMERICA

## Abstract

Glucosinolate are one of the vital secondary metabolites in alfalfa (*Medicago sativa* L.), and primarily present as β-D-glucosinolate derivatives, improving the resistance in response to biotic and abiotic stresses of alfalfa. CYP79 (Cytochrome P450 monooxygenases) and CYP83 gene families play an important role in the core structure biosynthesis of glucosinolate. Nevertheless, a comprehensive exploration of CYP79 and CYP83 family members in alfalfa has thus far not been study. The types of glucosinolate in alfalfa were qualitative and quantitative analysis by UPLC-MS/MS. Then, we identified *MsCYP79* and *MsCYP83* gene families in alfalfa, and scrutinized the physicochemical attributes, gene architecture, collinearity, evolutionary trajectories, as well as expression patterns under mechanical damage. The findings revealed the glucosinolate metabolites of alfalfa divided into three classes, including 27 aliphatic glucosinolates, 9 aromatic glucosinolates, and 5 indole glucosinolates. In addition, 59 *MsCYP79* family members and 56 *MsCYP83* family members were identified in alfalfa, which were classified into eight main groups based on phylogenetic analysis. *MsCYP79* and *MsCYP83* were distributed unevenly on 26 chromosomes and had 2–6 exons. Then, employing MEME software unveiled 15 conserved motifs within the protein structures of MsCYP79 and MsCYP83. Real-time quantitative PCR was used to detect the expression level of *MsCYP79* and *MsCYP83* genes and demonstrated that the selected genes in alfalfa were tissue-specific and had different expression patterns in response to mechanical damage. This investigation laid a robust groundwork for substantiating the functions of *MsCYP79* and *MsCYP83* and facilitating the cultivation of alfalfa varieties enriched in glucosinolate content.

## Introduction

Glucosinolate (GS), are a sulfur- and nitrogen-rich plant secondary metabolite, whose hydrolysis products have a wide range of biological activities, such as chemopreventive, antioxidative, antimicrobial, and antibacterial properties [1–3]. For GS, the regulatory functions in defensing against various environmental stresses and antimicrobial properties, have been extensively investigated and validated in *Arabidopsis* [4–6]. In addition, studies have shown that specific glucosinolate, such as glucoraphanin and glucobrassicin, as well as their degradation products, especially isothiocyanates, can induce the deactivation of carcinogens. Consequently, there is ongoing discourse regarding the consumption of glucosinolate-rich vegetables as a potential strategy to mitigate the risk of cancer formation [7,8]. In plants, glucosinolate generally divide into three groups based on based on the side-chain structure and the respective amino acid precursor: (a) aliphatic glucosinolate, (b) aromatic glucosinolate, and (c) indole glucosinolate [9,10]. To date, different classes of GS are presumed to play distinctive roles in mediating plant responses to various biotic and abiotic environmental stress factors.

The biosynthesis of GS can be divided into three independent stages: (1) chain elongation of selected precursor amino acids, (2) formation of the core structure, and (3) secondary modifications of the amino acid side chain [11]. The GS biosynthesis is involved in a large number of genes, enzymes, and transcriptional regulatory factors. The formation of the glucosinolate core structure seemed almost completely elucidated. Briefly, the first common step involves the cytochromes P450 (CYP450) of the CYP79 family, which catalyzes the conversion of amino acids into their corresponding aldoximes, while CYP83 family facilitates the transformation of aldoximes into GS (S1 Fig) [12]. Formation and conversion of the aldoxime is a key regulatory step in glucosinolate biosynthesis, thus, the identification of the *CYP79* and *CYP83* gene families catalyzing this process has provided indispensable tools to metabolically engineer functional food crops with improved nutritional value and improved insect and pathogen resistance [13]. Currently, the regulatory network of GS biosynthesis and metabolism in the model plant *Arabidopsis* has been elucidated. Firstly, amino acid precursors are catalyzed aldoxime by CYP79 family enzymes. For example, the *Arabidopsis* genome has seven *CYP79s*, among the characteristics of five enzymes have been extensively studied, CYP79A2 catalyzing phenylalanine, CYP79B2 and CYP79B3 catalyzing tryptophan, CYP79F1 catalyzing methionine 6, and CYP79F2 catalyzing methionine 5,6 [14,15]. Aldoximes are subsequently oxidized by CYP83 family enzymes to generate nitriles or aci-nitro compounds. In *Arabidopsis*, CYP83A1 is responsible for catalyzing aliphatic oximes, while CYP83A1 and CYP83B1 collaboratively catalyze aromatic oximes [13]. Notably, Zang *et al*. [16] demonstrated that the *Arabidopsis CYP83A1* gene was transferred to Chinese cabbage (*Brassica campestris* L. ssp. Pekinensis) to increase the accumulation ability of aliphatic glucosinolate. Meenu *et al*. [17] observed the aliphatic glucosinolate contents improved nearly double by transferring the *BjuCYP83A1-1* gene from *Capsella bursa pastoris* L. into *Arabidopsis*. In addition, Weis *et al*. [18] found that the content of aliphatic glucosinolate in the *CYP83A1* deficient mutant of *Arabidopsis* was significantly lower than that of the wild type. Furthermore, with the development of plant gene engineering, the members of the CYP79 and CYP83 families will be identified from an increasing number of plant species, so as to lay the foundation for confirming these gene functions via bioinformatics analysis and genetic transformation.

Alfalfa (*Medicago sativa* L.), as an important leguminous forage crop around the world, is widely and intensively cultivated because of high yield, exceptional quality, strong stress resistance, and wide adaptability [19,20]. However, *Odontothrips loti* of the dominant thrips species pose a severe threat to alfalfa production and nutritional value, in northern China, causing yield losses exceeding 30%. What is more, insect attack can induce a significant increase of the certain glucosinolate or total glucosinolate contents in the damaged plants, indicating the defensive role of glucosinolate against herbivorous insects [21,22]. Notably, aliphatic glucosinolate can resist the infestation of leaf-eating pests and some host pathogenic bacteria, but indole glucosinolate can effectively deter insect oviposition and non-host pathogenic bacteria [18,23]. Lu [24] demonstrated through KEGG enrichment analysis that genes regulating glucosinolate metabolism exhibited higher expression levels, suggesting a correlation between glucosinolate metabolism genes and aphid resistance. Therefore, cultivating new alfalfa cultivars that are resistant to insects and enriched with high glucosinolate content holds practical significance in meeting the production needs of alfalfa in China. In recent years, with the completion of alfalfa genome sequencing, using bioinformatics methods to analyze and identify the members of the CYP79 and CYP83 gene family has become an important approach for exploring functional genes involved in alfalfa glucosinolate biosynthesis. While research on the CYP79 and CYP83 gene families in *Arabidopsis* is relatively thorough, the analysis and identification of CYP79 and CYP83 family members in alfalfa is currently lacking by means of a genomic level.

In the study, we aimed to clarify the putative CYP79 and CYP83 genes involved in glucosinolate biosynthesis and mechanical damage, and provide potential candidate *CYP79* and *CYP83* genes for further explaining glucosinolate biosynthesis and regulation in alfalfa. We firstly investigated the glucosinolate content and category of alfalfa. Subsequently, using the latest available genome assembly and annotation database, we identified the members of the *CYP79* and *CYP83* gene family in the alfalfa genome and comprehensively analyzed. This analysis encompassed the sequence characteristics, gene structure, location, phylogeny, collinearity relationships, as well as expression patterns in different tissues and under mechanical damage. The research results of this study not only provide theoretical basis for understanding the functions and characteristics of the CYP79 and CYP83 family members in alfalfa, but also have important significance for employing molecular approaches to regulate glucosinolate biosynthesis of alfalfa.

## Materials and methods

### Plant materials

In this experiment, the seeds of the tested experiment alfalfa cultivar (*Medicago sativa* cv. Gannong NO.9, recorded as G9) were provided by the Key Laboratory of Grassland Ecosystem of the Ministry of Education. G9 was cultivated at Lanzhou Institute of Husbandry and Pharmaceutical Sciences of CAAS (36°05′N, 103°41′E, 1604 m altitude), Lanzhou, northwestern China. The indoor temperature was maintained at 25 °C ± 3 °C, with a photoperiod of 16 h of light and 8 h of darkness. Alfalfa seeds were sterilized with a 10% sodium hypochlorite solution, followed by rinsing with sterilized distilled water, and were then planted in plastic plots to germinate. Consistent and uniform growth of five seedlings per plot was achieved by watering with 100 mL every 2 ~ 3 d and 100 mL of full-strength Hoagland’s solution per week to ensure normal growth. After 45 d, alfalfa plants were divided into a mechanical damage treatment group and a control group (CK). The CK consisted of healthy plants without any treatment, and samples of roots, stems, and leaves samples were collected. For the mechanical damage treatments, a 2 mm diameter punch was used to create holes in alfalfa leaves, with 10 holes per leaf. In addition, samples of healthy, undamaged leaves (0 h) and leaves with mechanical damage were collected for 12 h, 24 h, 48 h, and 72 h. All samples were promptly frozen in liquid nitrogen and stored at -80°C. Three replicates were performed for each sample.

### Metabolomics analysis of glucosinolate

The qualitative analysis of glucosinolate in healthy alfalfa leaves was detected by MetWare (Wuhan, China, http://www.metware.cn/). The freeze-dried samples were ground at 30 Hz for 1.5 min by using a grinder (MM 400, Retsch). Next, 50 mg sample powder was added 1200 μL of -20 °C pre-cooled 70% mixture with methanol and internal standard extract, and vortexed once every 30 min for 30 s, for a total of 6 times. After centrifugation at 12000 rpm for 3 min, the supernatant was aspirated, and the sample was filtered through a 0.22 μm microporous membrane (SCAA-104, ANPEL, Shanghai) and stored in the injection vial for UPLC-MS/MS analysis (UPLC: SHIMADZU Nexera X2, https://www.shimadzu.com.cn/, MS/MS: Applied Biosystems 6500 QTRAP, http://www.appliedbiosystems.com.cn/). The column of UPLC conditions was Agilent SB-C18 (1.8 µm, 2.1 mm * 100 mm), as well as the mobile phase was consisted of solvent A (pure water with 0.1% formic acid) and solvent B (acetonitrile with 0.1% formic acid). The flow velocity, column temperature, and injection volume were set as 0.35 mL per minute, 40°C, 2 μL, respectively. The ESI source operation parameters were as follows: source temperature 500°C, ion spray voltage (IS) 5500 V (positive ion mode)/-4500 V (negative ion mode), ion source gas I (GSI), gas II(GSII), curtain gas (CUR) were set at 50, 60, and 25 psi, respectively. Instrument tuning and mass calibration were performed with 10 and 100 μmol/L polypropylene glycol solutions in QQQ and LIT modes, respectively. QQQ scans were acquired as MRM experiments with collision gas (nitrogen) set to medium. DP (declustering potential) and CE (collision energy) for individual MRM transitions was done with further DP and CE optimization. A specific set of MRM transitions were monitored for each period according to the metabolites eluted within this period (S2 and S3 Figs). Based on the MetWare database, qualitative analysis of the glucosinolate in the sample was conducted by Analyst 1.6.3 software and MultiQuant software.

### *MsCYP79* and *MsCYP83* gene identification

To identify *MsCYP79* and *MsCYP83* genes in alfalfa, the protein sequences of CYP79 and CYP83 from *Arabidopsis*, *Oryza sativa* and *Medicago truncatula* were retrieved and downloaded from the database (http://www.arabidopsis.org/, http://rice.uga.edu/, https://ftp.ncbi.nlm.nih.gov/genomes/all/GCA/000/219/495/GCA000219495.2_MetrA17_4.0/), and then used as the queries to perform a BLASTP search against the local protein database of alfalfa, which was downloaded from database (https://figshare.com/projects/whole_genome_sequencing_and_assembly_of_ Medicago_sativa/66380) [25]. Meantime, the typical domain sequences of *CYP79* and *CYP83* (PF00067) were downloaded from the PFAM database (http://pfam.xfam.org/), and the proteins with this domain in the local alfalfa protein database were identified using HMMER 3.0 software. Furthermore, protein sequences identified by both methods underwent manual parsing by to eliminate redundancy. The resultant proteins were considered candidate alfalfa *CYP79* and *CYP83* proteins.

### Physical and chemical properties of proteins and chromosomal localization analysis

The amino acid residue, molecular weights (MWs), theoretical isoelectric points (pIs), and grand average of hydropathicity (GRAVY) values for MsCYP79 and MsCYP83 proteins were calculated using the online ExPASy program (http://web.expasy.org/protparam/). Subcellular localization of MsCYP79 and MsCYP83 proteins was predicted using the ProtComp 9.0 (http://linux1.softberry.com/berry.phtml?topic=index&group=programs&subgroup=proloc). The chromosome distribution of *MsCYP79* and *MsCYP83* genes was investigated using MG2C v2.1 software (http://mg2c.iask.in/mg2c_v2.1/).

### Gene structure analysis and promoter region cis-regulatory elements

The deduced amino acid sequences of MsCYP79 and MsCYP83 were analyzed using the MEME v5.0.5 program, with a maximum motif count set to 15 (http://meme-suite.org/) for conserved motif identification. Chromosomal location and exon-intron structures of these *MsCYP79* and *MsCYP83* genes were extracted from genome annotation files downloaded from the Ensembl Plants database. The gene structure and intron phases were visually presented using the Gene Structure Display Server (http://gsds.cbi.pku.edu.cn/) based on the genomic sequences and corresponding coding sequences (CDSs).

### Phylogenetic analysis of the MsCYP79 and MsCYP83

To unravel the evolutionary relationship of the alfalfa MsCYP79 and MsCYP83 genes, we conducted multiple sequence alignments incorporating identified MsCYP79 and MsCYP83 proteins from alfalfa and model plants, such as *Arabidopsis thaliana*, *Oryza sativa*, and *Medicago truncatula*. The phylogenetic tree was performed using the ClustalW tool, and a neighbor-joining (NJ) tree was constructed via MEGA v10 software (https://www.megasoftware.net/), employing a bootstrap value of 1000.

### Analysis of promoter cis-acting elements

Promoter sequences spanning 2.0 kb for *MsCYP79* and *MsCYP83* genes were examined to identify cis-acting elements in the promoter regions using the Plantcare website (http://bioinformatics.psb.ugent.be/webtools/plantcare/html/).

### Real-Time PCR gene expression analysis

Total RNA extraction from leaves of control and treated seedlings was performed using TransZol reagent (Tiangen, Beijing, China). Reverse transcription and cDNA synthesis were conducted using a Hifair^®^ Ⅲ 1st Strand cDNA Synthesis SuperMix for qPCR (Yeasen Biotech Co., Ltd., Shanghai, China) from 1 µg of total RNA. qRT-PCR was executed on each cDNA template using Hieff® qPCR SYBR Green Master Mix (Low Rox Plus) (Yeasen Biotech Co., Ltd., Shanghai, China) on a CFX96 Real-Time PCR Detection System (Bio-Rad, Los Angeles, CA, USA). Primer sets are listed in S3 Table. In this study, *18S rRNA* was as an internal reference gene. Expression levels were calculated relative to controls and determined using the 2^-^^△△^^CT^ method [26,27]. Each of the three biological replicates was supported by three technical replicates.

### Glucosinolate content

Total glucosinolate contents were determined using a glucosinolate detection kit (SolarbioBiotech Co. Ltd., Beijing, China), with four biological replicates for each sample. Briefly, Freeze-dried sample powder (0.1 g) and distilled water (1 mL) were mixed in a 2-mL tube, and extracted with water bath at 95 °C for 30 min. Afterward, the sample was centrifuged at 10,000 × g under 4 °C for 10 min when sample cooled down to room temperature, the 50 μL supernatant was collected and used for total glucosinolate analysis. Then we added 100 μL reagent one, 50 μL reagent two and kept it at room temperature for 2 h. Finally, we measured the absorbance of the reaction in A540.

## Results

### Glucosinolate type

This study conducted both qualitative analyses of glucosinolate in alfalfa (*Medicago sativa* cv. Gannong NO.9) (S2 and S3 Figs). The detailed classification of glucosinolate in alfalfa was presented in Table 1. The results identified the 41 glucosinolates, including three classes: 27 aliphatic glucosinolates, 9 aromatic glucosinolates, and 5 indole glucosinolates. Therefore, these results indicated that the aliphatic glucosinolate was predominant category. Furthermore, the quantitative analysis indicated that the total relative content of aliphatic glucosinolate was significantly higher than that of indole glucosinolate, while the total relative content of aromatic glucosinolate was the lowest. Among the 41 glucosinolates, the relative contents of 1-hydroxyindole-3-methylthioglucosinolate and 4-hydroxyindol-3-ylmethyl glucosinolate were generally much higher than those of other glucosinolates. Additionally, qualitative analysis showed that all three glucosinolates belong to the indole glucosinolate category.

**Table 1:** The classification and relative content of glucosinolate.

Classes	Compounds	Relative content
aliphatic glucosinolate	4-Methylsulfonyl-3-butenyl Glucosinolate	26528.95
	5-Hexenyl Glucosinolate	38647.86
	Hexyl Glucosinolate*	14674.10
	6-Heptyl Glucosinolate	11926.53
	7-(Methylsulfinyl)Heptyl Glucosinolate	277440.49
	7-Methylthioheptyl Glucosinolate*	63940.96
	6-Methylsulfinylhexyl Glucosinolate	232489.18
	5-Methylsulfinylpentyl glucosinolate	59420.35
	2(R)-Hydroxy-3-butenyl glucosinolate	28274.90
	2-Pentanone glucosinolate	59189.17
	4-(Methylsulfonyl)butyl glucosinolate	20869.29
	8-(Methylsulfinyl)octenyl thioglycoside	54207.23
	4-(2-amino-3-carboxyethylthio) butyl glucosinolate	157247.87
	5-(Methylthio)butyl glucosinolate	78151.72
	8-(Methylthio)heptyl glucosinolate*	60961.38
	9-(Methylthio)octyl glucosinolate	15274.71
	10-(Methylthio)nonylthioglucosinolate	33287.64
	Heptyl glucosinolate	11926.53
	7-Octenyl thioglycoside	38894.73
	4-Methylsulfinyl-3-Butenyl Glucosinolate	9219.77
	6-Methylthiohexyl glucosinolate (Glucolesquerellin)	60245.48
	8-Methylthiooctyl glucosinolate	53141.46
	Sinigrin (2-Propenyl Glucosinolate)	62907.07
	4-Methylsulfinylbutyl glucosinolate (Glucoraphanin)	18167.99
	Glucocheirolin	15964.77
	4-Methylamyl Glucosinolate*	9574.41
	2-Hydroxy-2-methylpropylglucosinolate	208581.31
aromatic glucosinolate	2(R)-Hydroxy-2-Phenylethyl Glucosinolate	44712.33
	2-benzoyloxy-1-ethyl glucosinolate*	56932.20
	o-coumaric acid glucosinolate*	218709.55
	p-coumaric acid glucosinolate*	228502.43
	4-Phenylbutyl glucosinolate	12481.83
	Benzoyloxy butyl glucosinolate*	29264.93
	Benzoyloxy amyl glucosinolate	13096.29
	2-benzoyloxy-3-butenyl glucosinolate	37388.33
	2-(4-Methoxyphenyl)-2,2-dimethyl ethyl glucosinolate	23726.45
indole glucosinolate	1,4-Dimethoxyglucobrassicin	2383.50
	1-hydroxyindole-3-methylthioglucosinolate	571386.24
	1-Methoxy-3-indolylmethyl glucosinolate (Neoglucobrassicin)	79261.72
	4-Hydroxyindol-3-ylmethyl glucosinolate	452611.70
	3-Indolylmethyl glucosinolate	74017.53

### Identification and analysis of MsCYP79 and MsCYP83 family

In the alfalfa genome, we eventually discovered 59 *CYP79* and 56 *CYP83* family genes, which were designated as MsCYP1 to MsCYP115 based on their sequence similarity and the phylogenies relationships of CYP79 and CYP83 proteins in *Arabidopsis thaliana*, *Oryza sativa*, and *Medicago truncatula*. Amino acid count, CDS length, molecular weight (MW), isoelectric points (PI), and GRAVY of the *MsCYP79* and *MsCYP83* proteins were selected for further analyses (S1 Table). The length of the deduced MsCYP79 and MsCYP83 protein sequences varied from 201 to 539 amino acids. Among them, the MsCYP51, MsCYP90 of MsCYP83 comprised only 201 amino acids and weighed 5.44 and 6.84 kDa, respectively. In contrast, the MsCYP33, MsCYP4, MsCYP67, and MsCYP74 of MsCYP79 had 539 amino acids, with protein molecular weights of 8.88, 9.02, 8.88, and 8.86 KDa, respectively. The gene sequence lengths of *MsCYP79* and *MsCYP83* families exhibited considerable diversity, which changed between 606 bp and 1620 bp. Among them, *MsCYP33*, *MsCYP4*, *MsCYP67*, and *MsCYP74* of *MsCYP79s* had the longest sequence (1620 bp), followed by *MsCYP34* of *MsCYP79s* (1611 bp). Conversely, *MsCYP51, MsCYP90* of *MsCYP83s* had the shortest gene sequence (606 bp). There were 57 *MsCYP79* genes and 36 *MsCYP83* genes with gene sequence lengths exceeding 1000 bp. The isoelectric points of the 10 MsCYP79 proteins (MsCYP108, MsCYP110, MsCYP30, MsCYP36, MsCYP6, MsCYP69, MsCYP75, MsCYP77, MsCYP79, MsCYP8) were below 7, indicating that the 10 proteins were acidic, whereas the remaining 49 MsCYP79 proteins were alkaline. However, the isoelectric points of the 4 MsCYP83 proteins (MsCYP101, MsCYP58, MsCYP64, MsCYP65) were above 7, suggesting them were alkaline, while the remaining 52 MsCYP83 proteins were acidic. The hydrophobicity index (GRAVY) of MsCYP79 and MsCYP83 proteins was less than 0, indicating that these were all hydrophilic proteins. Subcellular localization predictions revealed that MsCYP79 proteins were predominantly localized in the endoplasmic reticulum, while MsCYP83 proteins were mainly localized in the plasma membrane.

### Chromosome location and collinear analyses

**Fig 1:**
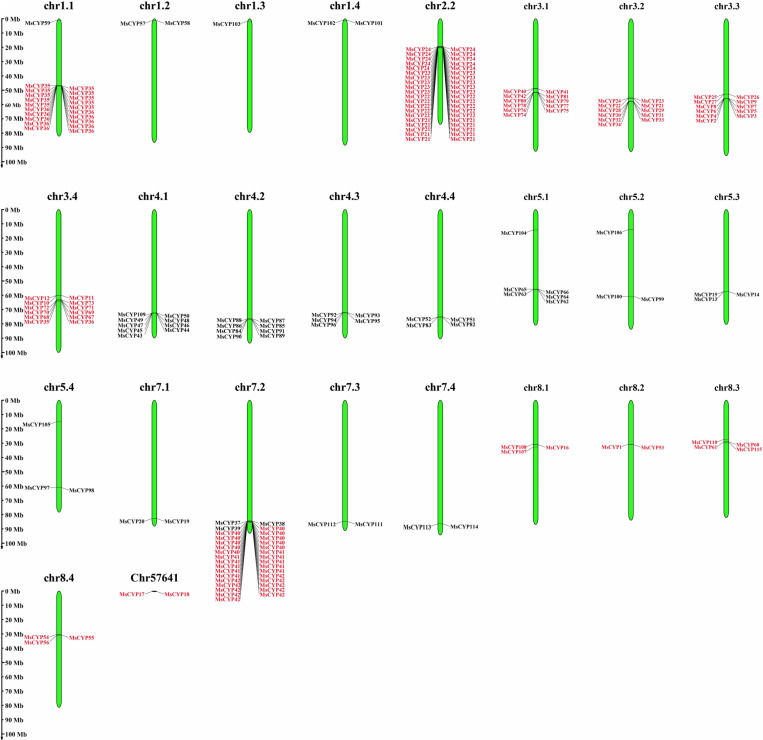
Location of *MsCYP79* and *MsCYP83* family genes in alfalfa.

**Fig 2:**
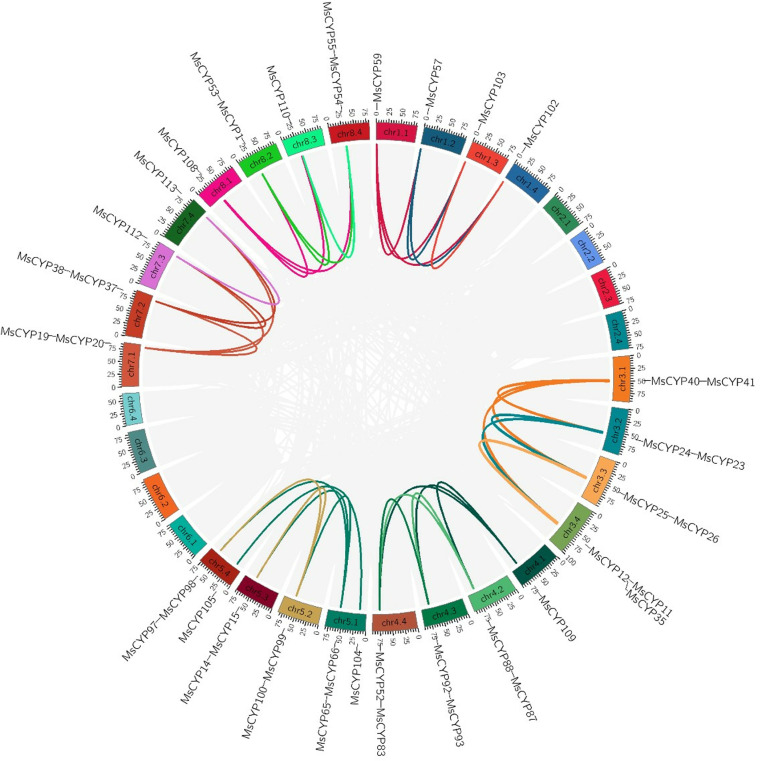
Collinearity of *MsCYP79* and *MsCYP83* gene family in alfalfa.

Both tandem duplication and segmental duplication contribute to the generation of gene family during the progress of evolution. To further investigate the evolutionary mechanism of *MsCYP79* and *MsCYP83* family genes, the genomic position were analyzed via MapGene2Chrom software. The distribution of *MsCYP79* and *MsCYP83* family genes were unevenly in the 26 chromosomes of alfalfa (Fig 1). Among them, there were 12 genes on the chr3.4, representing the most abundant regions, followed by chr3.1, chr3.2, chr3.3 with 11 genes, while chr1.3 had the only 1 gene. In addition, the *MsCYP79* genes exhibited significant tandem repeats, and presented four clusters of tandem repeats on chromosomes. *MsCYP21*, *MsCYP22*, *MsCYP23*, and *MsCYP24* formed a large tandem repeat gene cluster on chr2.2, and *MsCYP40*, *MsCYP41*, and *MsCYP42* formed a large tandem repeat gene cluster on chr7.2. 99 pairs of collinear relationships within *Medicago sativa* (Fig 2).

**Fig 3:**
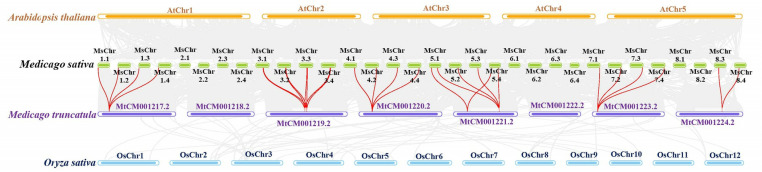
Collinearity analysis of the *CYP79* and *CYP83* genes between *Medicago sativa* and *Arabidopsis thallana*, *Medicago truncatula*, *Oryza sativa.*

Furthermore, four comparative syntenic maps of *Medicago sativa*, with three representative plant species *Arabidopsis*, *Oryza sativa*, *Medicago truncatula* were constructed to illustrate the evolution mechanism of *MsCYP79* and *MsCYP83* family genes (Fig 3). The results highlighted that the 28 homologous pairs were observed between *Medicago sativa* and *Medicago truncatula*, but no homologous pairs were found between *Medicago sativa* and *Arabidopsis*, *Oryza sativa*.

### Gene structure and motif composition

**Fig 4:**
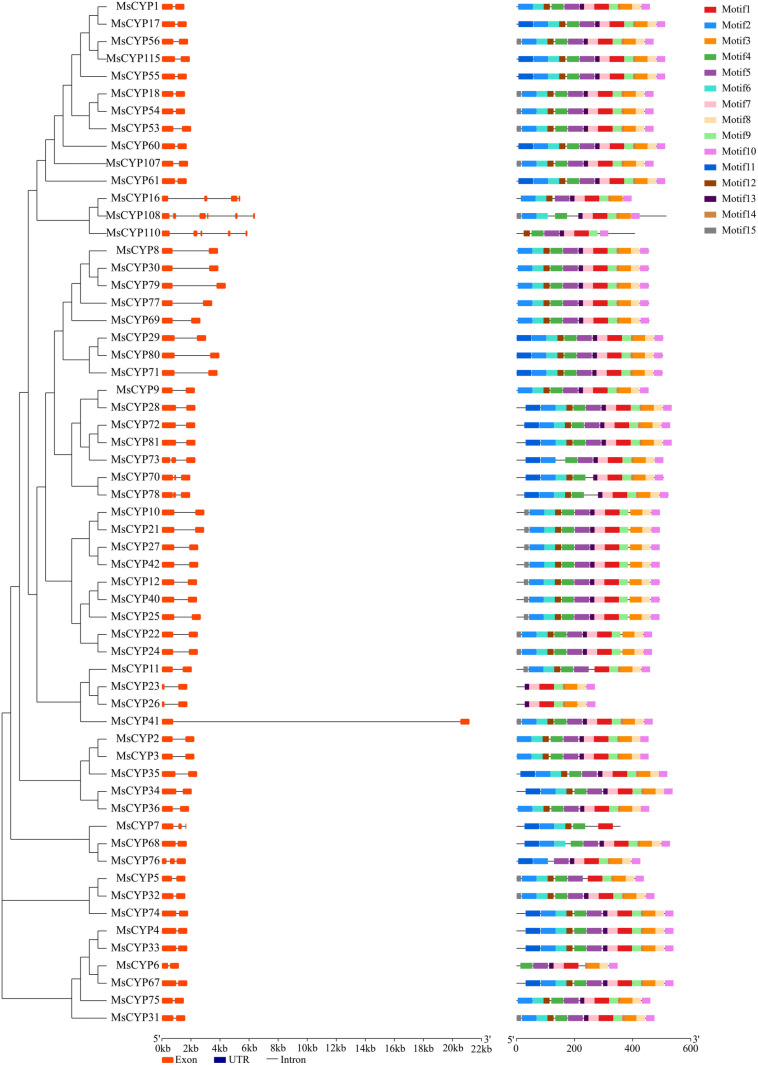
Gene structure and motifs of *MsCYP79* in alfalfa.

**Fig 5:**
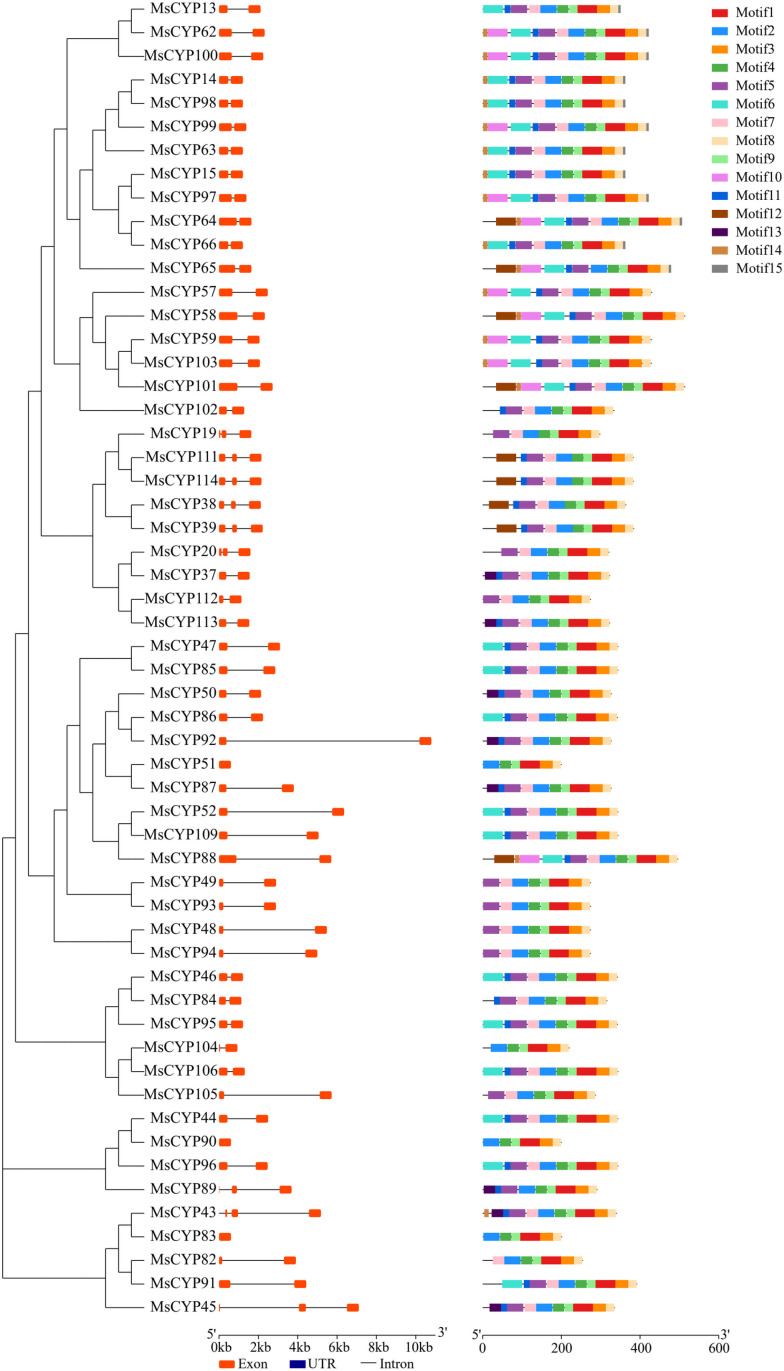
Gene structure and motifs of *MsCYP83* in alfalfa.

To further demonstrate the structural features of *MsCYP79* and *MsCYP83* family genes, we conducted a comparative analysis of the intron-exon structures according to their evolutionary relationships. Results showed that the coding sequences of *MsCYP79* and *MsCYP83* family genes were interspersed with introns ranging from 0 to 5 (Fig 4, and Fig 5). Among them, 51 *MsCYP79* and 44 *MsCYP83* family members had only 1 intron, implying their close phylogenetic relationship. On the other hand, *MsCYP51*, *MsCYP83*, and *MsCYP90* of *MsCYP83* family genes featured a single exon with no introns, while some members displayed 5 introns and 4 introns, for examples, *MsCYP108*, *MsCYP110* of *MsCYP79* family genes, respectively.

**Fig 6:**
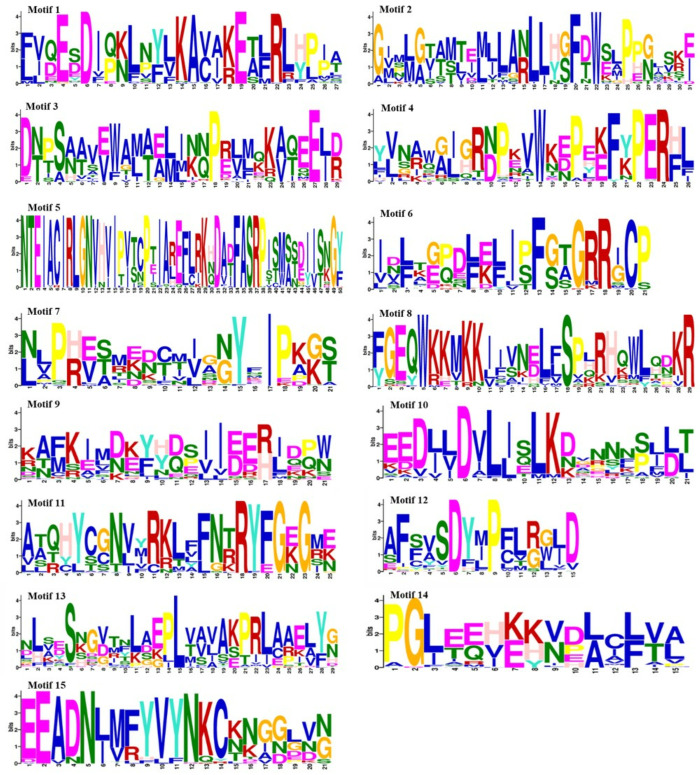
Sequence of MsCYP79 and MsCYP83 motifs in alfalfa.

Then, the conserved motifs in the *MsCYP79* and *MsCYP83* family genes were predicted by MEME motif analysis. We identified the 15 conserved motifs in MsCYP79 and MsCYP83 proteins, namely motif1 ~ motif15 (Table 2, Fig 6). It showed that the 41 members of the MsCYP79 proteins contained all these 15 motifs, indicating that these MsCPY79 protein members may function similarly. However, only 11 members of MsCYP83 proteins and 3 members (MsCYP23, MsCYP26, MsCYP7) of MsCYP79 proteins had less than 10 motifs. The distribution of protein motifs in MsCYP79 and MsCYP83 established a foundation for phylogenetic analysis.

**Table 2:** Motif sequences for predicting MsCYP79 and MsCYP83 proteins.

NO.	Motif sequence	Width
1	FVQESDIQKLNYLKAVAKETLRLHPIA	27
2	GIMLGTAMTEMJLANLLHGFDWSLPPGLSKE	31
3	DNPSAAVEWAMAELINNPRLMQKAQEEJD	29
4	YVNAWAJGRBPKVWKEPEEFYPERHL	26
5	NTEIACIRLGNVHVIPVTCPTIAREFLRKHDADFASRPISMSSDIISNGY	50
6	IDLTGPDLELIPFGTGRRICP	21
7	NLPHESMEDCMIGNYFIPKGS	21
8	FGEQWKKMKKIIVNELFSPLRHQWLQBKR	29
9	KAFKIMBKYHDSIIEERJDPW	21
10	EEDJLDVLJSLKDGNNNSJLT	21
11	ATQHYCGNVYRKLFFNTRYFGEGME	25
12	AFSVSDYMPFLRGJD	15
13	BLSESNGVTNLAEPLVAVAKPRLAAELYG	29
14	PGLEZHEKVDLCLVA	15
15	EEADNJMFYVYNKCKNGGLVN	21

### Phylogenetic analysis and classification

**Fig 7:**
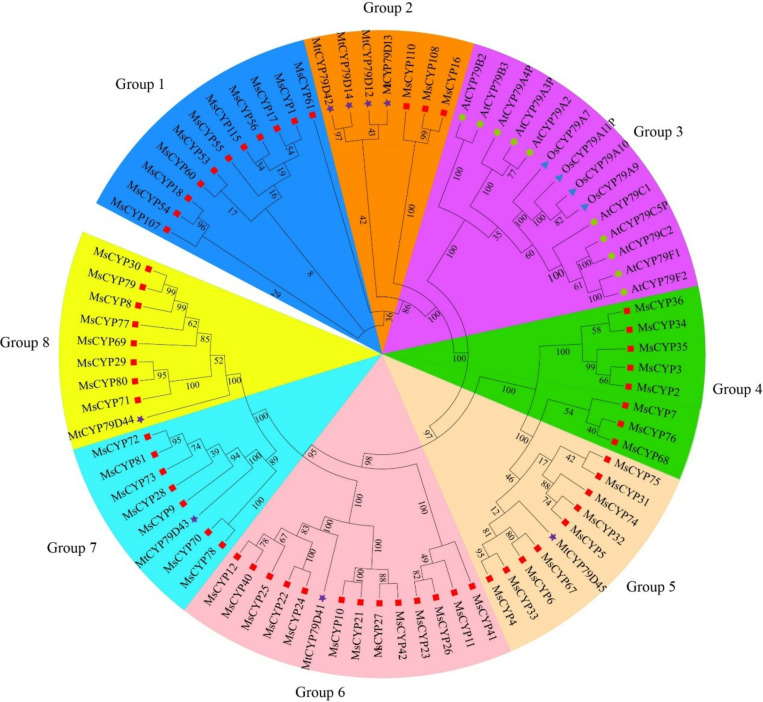
Phylogenetic tree of *MsCYP79* family in alfalfa.

**Fig 8:**
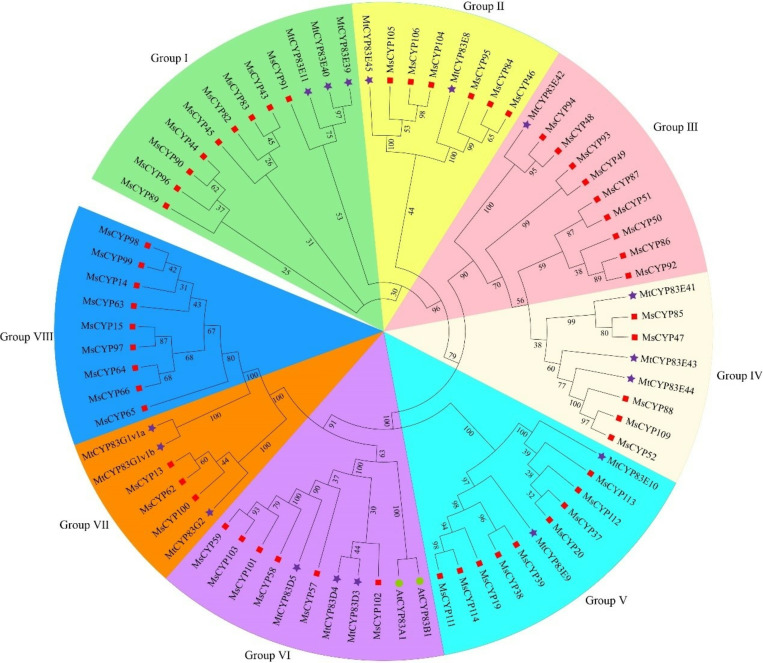
Phylogenetic tree of *MsCYP83* family in alfalfa.

In this study, to better understand *MsCYP79* and *MsCYP83* family genes evolutionary relationship, we constructed a phylogenetic tree using the NJ method, incorporating protein sequences from 12 AtCYP79 and AtCYP83 of *Arabidopsis thaliana*, 25 MtCYP79 and MtCYP83 of *Medicago truncatula*, 4 OsCYP79 of *Oryza sativa*, and 59 MsCYP79 and 56 MsCYP83 of *Medicago sativa*. As shown in Fig 7 and Fig 8, *MsCYP79* and *MsCYP83* family genes were unevenly clustered into 8 major branches, respectively. *MtCYP79* genes were distributed across Groups 1, 2, 4, 5, 6, 7, and 8, while Group 3 did not include any members from *Medicago sativa*, featuring only *AtCYP79* and *OsCYP79* genes. At the same time, in the phylogenetic tree of *MsCYP83* genes, Groups Ⅰ, Ⅱ, Ⅲ, Ⅳ, Ⅴ, Ⅵ, Ⅶ, and Ⅷ comprised nine, six, nine, five, nine, six, three, and nine genes, respectively. Notably, *AtCYP83A1*, and *AtCYP83B1* genes were exclusively present in Group Ⅵ.

### Analysis of promoter cis-acting elements

To further investigate the transcriptional regulation mechanism and potential functions of *MsCYP79* and *MsCYP83* genes, the cis-regulatory elements of the promoter were predicted. As shown in S2 Table, totally, 12 functional cis-elements were obtained, of which CAAT box, TATA box were found in all of *MsCYP79* and *MsCYP83* genes. Meanwhile, the promoter region of *MsCYP79* and *MsCYP83* contained regulatory elements essential for anaerobic induction (ARE), light responsiveness (G-box, Box 4, GT1-motif, GATA motif, etc.).

### Expression patterns of MsCYP79 and MsCYP83 genes in different tissues

**Fig 9:**
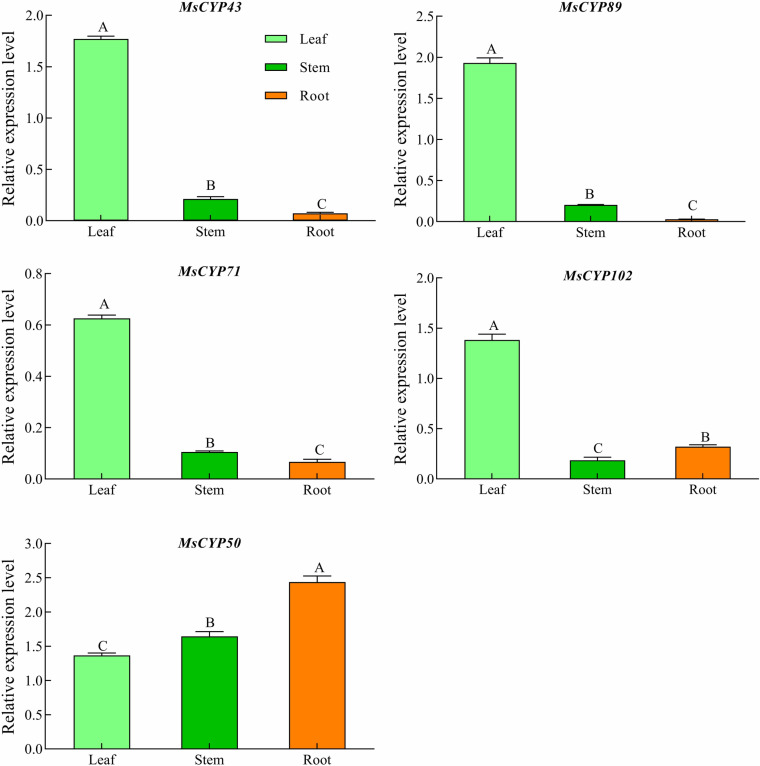
Relative expression of *MsCYP79* and *MsCYP83* genesin different tissues of alfalfa. Note: Different capital letters indicated significant difference of the different tissues in *Medicago sativa* cv. Gannong NO.9 at *P < 0.05* level.

To gain more insight into the role of *MsCYP*79 and *MsCYP83* genes in alfalfa, we analyzed through RT-qPCR the expression levels of *MsCYP*79 and *MsCYP83* genes in major tissues, including leaves, stems, and roots (Fig 9). Among them, the relative expression levels of *MsCYP43*, *MsCYP89*, and *MsCYP71* were highest in leaves, followed by stems, and were lowest in roots. Conversely, the *MsCYP50* relative expression level was highly expressed in roots, followed by stems, and had lowest expressions in leaves. In addition, *MsCYP102* exhibited the highest relative expression in leaves, followed by roots, and was lowest in stems.

### Expression patterns of MsCYP79 and MsCYP83 genes under mechanical damage

**Fig 10:**
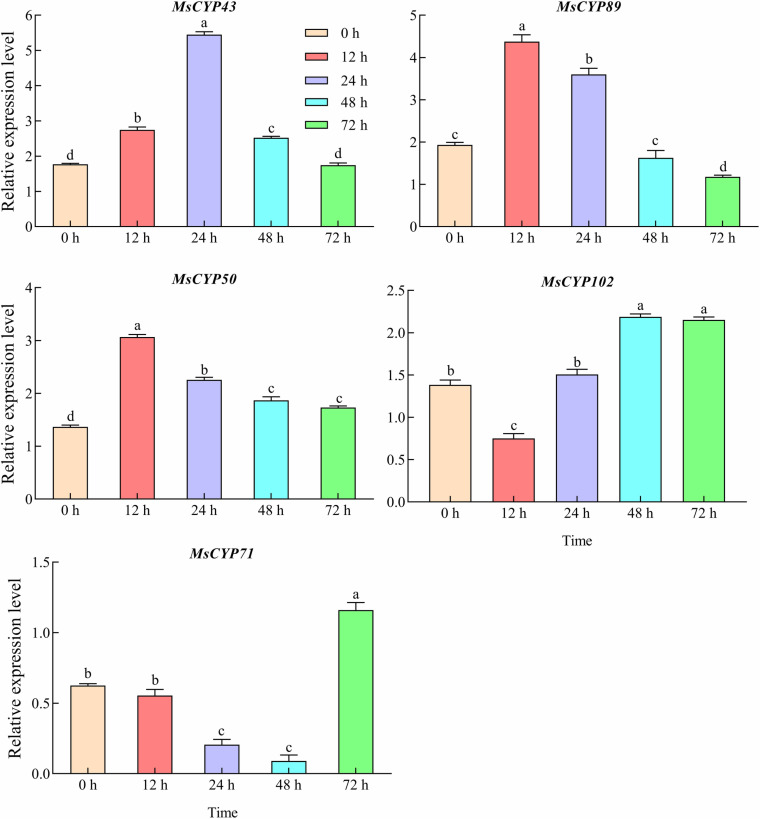
Relative expression of *MsCYP79* and *MsCYP83* genes of alfalfa under mechanical times. Note: Different small letters indicated significant difference of the *Medicago sativa* cv. Gannong NO.9 under mechanical damage treatment at *P* < 0.05 level.

We determined the expression patterns of *MsCYP79* and *MsCYP83* genes in *Medicago sativa* cv. Gannong NO.9 under mechanical damage by RT-qPCR (Fig 10). Interestingly, the relative expression levels of *MsCYP43*, *MsCYP89*, and *MsCYP50* exhibited an initial upregulation followed by a decline, reaching their highest levels at 24 h, 12 h, 12 h, respectively. In contrast, the relative expression level of *MsCYP102* showed a downward-rising-decreasing trend with increasing mechanical damage time, reaching a peak at 48 h. Mechanical damage treatment induced *MsCYP71* to reach its highest levels at 72 h, representing an 85.36% increase compared to 0 h.

### Glucosinolate content of the alfalfa in different tissues and mechanical damage

**Fig 11:**
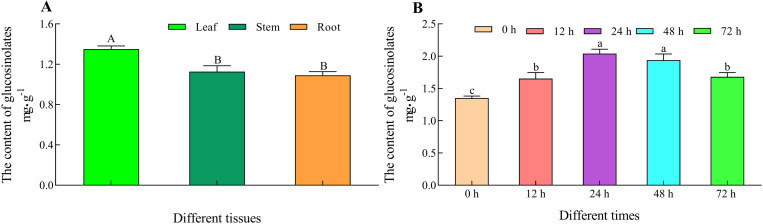
The glucosinolates content of alfalfa.

Analysis of the total glucosinolate contents in alfalfa revealed that the highest glucosinolate content was observed in the leaves, followed by that in the stems, and the lowest was observed in the roots (Fig 11A). The glucosinolate content in leaves were significantly higher than the stem (19.92%) and roots (24.00%) (*P < 0.05*). In addition, the glucosinolate content change of the different tissues in alfalfa were consistent with the expression levels of *MsCYP43*, *MsCYP89,* and *MsCYP71.* Under mechanical damage, the glucosinolate contents of the leaves in G9 increased first but then decreased with increasing of the damage time (Fig 11B). Meanwhile, the glucosinolate contents had risen to the peak at 24 h, which was significantly increased by 51.00%, 23.42%, 21.43%, compared with 0 h, 12 h, 72 h (*P < 0.05*). Then, the glucosinolate content at 24 h were consistent with the *MsCYP43* expression levels.

## Discussion

As a secondary metabolite produced during the growth and development of plant, glucosinolate are well-known for their role in plant resistance to insects and pathogens, as well as for imparting special flavors to plants [28,29]. They are largely found in the order Brassicales, such as oilseed rape (*Brassica napus*), cabbage (*Brassica oleracea*), broccoli (*Brassica oleracea* var. *italica*), and the model plant *Arabidopsis thaliana* [4,30–32]. In a previous study, it was reported that the principal type of glucosinolate in *Brassica rapa* was aliphatic glucosinolate [33]. Wang *et al.* [34] had shown that aliphatic glucosinolate was the key metabolites that caused the difference in Chinese cabbage’s glucosinolate. In our study, the qualitative analysis revealed that 41 glucosinolates were detected in alfalfa, comprising 27 aliphatic glucosinolates, 9 aromatic glucosinolates, and 5 indole glucosinolates, this result signified that aliphatic glucosinolate was the main type of glucosinolates in alfalfa. Therefore, identification of glucosinolate types could be helpful in an effective marker-assisted breeding strategy for evaluating the function of genetic resources, and improving the resistance in alfalfa.

The biosynthesis of glucosinolate have been widely studied in model *Arabidopsis thaliana* [11,32], which *AtCYP79* and *AtCYP83* family genes is the vital component of glucosinolate biosynthesis, and plays the indispensable roles in its growth and development [35]. Recently, *CYP79* and *CYP83* family genes in various species is investigated [16,36–38], for example, 9 *CYP79* family members were identified in flax (*Linum usitatissimum* L.) [39]. However, little is known about the identification of *CYP79* and *CYP83* family genes in alfalfa. For this reason, this study focused on providing comprehensive information on the *MsCYP79* and *MsCYP83* family genes in alfalfa, which would provide a research basis for further studying function of *MsCYP79* and *MsCYP83* and genetic improvement of alfalfa. In this study, based on the genomic analysis, we identified 59 *MsCYP79* and 56 *MsCYP83* family genes of alfalfa (*Medicago sativa*), as well as 10 *AtCYP79s* and 2 *AtCYP83s* in *Arabidopsis thaliana*, 8 *MtCYP79s and* 17 *MtCYP83s* in *Medicago truncatula*, 4 *OsCYP79s* in *Oryza sativa*. The results proved that there were significant differences in the number of *CYP79* and *CYP83* family members among different species, which was related to gene deletion and expansion, genome size in the evolution of different species. A previous study reported that subcellular localization of the proteins was correlated with the protein *pI* values, and that the isoelectric points of cytosolic proteins fall below 7 [40]. In this study, the isoelectric points of 49 MsCYP79 proteins were greater than 7, which were located in the endoplasmic reticulum, indicating that played a crucial role in protein synthesis, folding, modification, and so on. In addition, the isoelectric points of 52 MsCYP83 proteins were blow 7, and subcellular localization showed that mainly localized in the plasma membrane, suggesting that developed a critical role in signal transduction, substance transport, and immune response etc. Based on the phylogenetic tree analysis, it can predict the function of genes in alfalfa by utilizing the functional characteristics of homologous genes from different species. Subsequently, the phylogenetic tree showed that *MsCYP79* and *MsCYP83* genes from alfalfa were classified into 8 groups, respectively.

Gene replication is an important mechanism for creating new genes and increasing genetic diversity [41,42]. In addition, gene replication can also expand the size of gene families and diversify gene functions [43]. Our research indicated that 59 *MsCYP79* and 56 *MsCYP83* genes were unevenly distributed on 26 alfalfa chromosomes, as well as the presence of tandem repeats in *MsCYP79* genes were found on chr 1.1, 2.2, and 7.2, which revealed that tandem duplications contributed to the evolution of *MsCYP79* genes in alfalfa. Furthermore, the results of collinearity analysis showed that 28 homologous pairs were observed between *Medicago sativa* and *Medicago truncatula*. The collinearity between the *MsCYP79* and *MsCYP83* gene of *Medicago sativa* and *Medicago truncatula* was significantly higher than that between *Medicago sativa* and *Arabidopsis thaliana*, *Oryza sativa*, possibly because *Medicago sativa* and *Medicago truncatula* were both leguminous plants.

The examination of promoter regions revealed that the *MsCYP79* and *MsCYP83* genes encompassed fundamental core elements, including the CAAT-box, TATA-box, and stress-related cis-acting elements such as ABRE, ARE, Box4, G-box, and GT1-motif. This observation suggested that *MsCYP79* and *MsCYP83* not only catalyzed the glucosinolate biosynthesis, but also actively participated in the regulation of multiple physiological processes, encompassing alfalfa’s response to hormones and abiotic stress. Moreover, it implied that the expressions of the *MsCYP79* and *MsCYP83* genes were intricately governed by various factors. Additionally, numerous studies have robustly demonstrated that MYB and MYC transcription factors serve as pivotal regulators of plant secondary metabolism, particularly in flavonoid and phenolic biosynthesis [44–47], implying that certain *MsCYP79* and *MsCYP83* genes may play a crucial role in the synthesis of flavonoids and phenolics. In this study, a significant proportion of *MsCYP79* and *MsCYP83* genes in alfalfa were found to harbor a notable number of MYB and MYC binding sites, which was crucial for exploring the transcription factors related to glucosinolate biosynthesis.

Previous research had indicated the involvement of certain *CYP79* and *CYP83* genes in response to insect pests and diseases [48,49]. *CYP83B1* has been studied very clearly in *Arabidopsis thaliana*, which mainly participates in the core structure formation of indole glucosinolate and can efficiently catalyze IAOx derived from tryptophan [50]. In the *CYP83B1* knockout plants, the excess indole-3-acetaldoxime was channeled into IAA biosynthesis, which led to elevated IAA and thus increased apical dominance, and reduced indole glucosinolate levels [2]. Conversely, overexpression of *CYP83B1* in *Arabidopsis thaliana* led to a reduced IAA, loss of apical dominance, but elevated indole glucosinolate levels [51]. These research results indicated that *CYP83B1* not only played a role in the biosynthesis of indole glucosinolate, but also affected the biosynthesis of IAA. Analyzing the expression patterns of gene family members across various plant tissues provides a more intuitive understanding of their functions. To investigate deeply into the roles of *MsCYP79* and *MsCYP83* family members, we conducted an expression analysis of *CYP83B1* (*MsCYP43, MsCYP50, MsCYP89*), *CYP71B* (*MsCYP102*) as well as isoleucine N-monooxygenase 2 (*MsCYP71*) in different alfalfa tissues. The relative expression levels of *MsCYP43*, *MsCYP89*, *MsCYP71*, *MsCYP102*, and *MsCYP102* exhibited distinct tissue-specific expressions and varied expression trends. In addition, the glucosinolate content change of the different tissues in alfalfa were consistent with the expression levels of *MsCYP43*, *MsCYP89*, and *MsCYP71*, which were positively correlated between glucosinolates content and *MsCYP43*, *MsCYP89*, *MsCYP71*. Research showed that mechanical damage and insect feeding could affect the glucosinolate content in plants [52,53]. Tian *et al*. [54] studied that the contents of total glucosinolates, aliphatic glucosinolates and indole glucosinolates in *Arabidopsis thaliana* were significantly increased at 3 h after mechanical wounding. In this study, the total glucosinolates contents of leaves in alfalfa were significantly improved under mechanical damage, compared with the undamaged leaves. Moreover, our findings revealed significant expression changes for *MsCYP43*, *MsCYP89*, *MsCYP71*, *MsCYP102*, and *MsCYP102* in response to mechanical damage. What is more, the glucosinolate content and *MsCYP43* expression level at mechanical damage time were positively correlated. These results suggested the potential involvement of these genes in biotic stress responses. To sum up, *MsCYP43* of *MsCYP83* family members was likely involved in glucosinolate biosynthesis and in response to mechanical damage.

In summary, the *CYP79* and *CYP83* family genes are prevalent gene families in plant, exhibiting diverse functions and significant regulatory effects on plant growth, development, and environmental adaptability. Despite their well-established roles, targeted research on these family genes in alfalfa remains unexplored, necessitating further investigations into their specific functions, transport substrates, and stress resistance mechanisms in future study.

## Conclusion

To sum up, this study represents the first comprehensive identification and analysis of the glucosinolate classes, and the *MsCYP79*, *MsCYP83* family genes in alfalfa. The aliphatic glucosinolate was predominant category in alfalfa. A total of 59 *MsCYP79* and 56 *MsCYP83* genes were identified, and their phylogenetics, chromosomal location, gene structure, and structural domains were systematically analyzed. Members of the *MsCYP79* and *MsCYP83* gene family members, such as *MsCYP43*, *MsCYP89*, *MsCYP71*, *MsCYP102*, *MsCYP50*, exhibited different spatiotemporal expression patterns in different tissues and mechanical damage time in alfalfa.

## Supporting information

S1 Table Analysis of physicochemical property of the MsCYP79 and MsCYP83 family genes. (PDF)

S2 Table cis‐acting elements in the promoter region of MsCYP79 and MsCYP83 family genes. (PDF)

S3 Table Primers used for RT-qPCR analysis. (PDF)

S1 Fig The biosynthesis pathway of glucosinolate. (TIF)

S2 Fig MRM detection of multimodal maps-Positive ion mode. (TIF)

S3 Fig MRM detection of multimodal maps-Negative ion mod. (TIF)

S4 Fig The gene expression levels of leaves in *Medicago sativa* cv. Gannong NO.9.
(TIF)
