# Supplementary material for: Identification of MsCYP79 and MsCYP83 gene families and its response to mechanical damage in Medicago sativa L
Source: PLoS One. 2025 May 8;20(5):e0322981. doi: 10.1371/journal.pone.0322981 (PMC12061124; doi:10.1371/journal.pone.0322981)
Supplement: S1 Table — (PDF) [file pone.0322981.s001.pdf]

S1 Table Analysis of physicochemical property of the MsCYP79 and MsCYP83 family genes

	Gene name	Gene ID	Number of animo acids (aa)	CDS length	Molecular weight (KDa)	Isoelectric point (pI)	GRAVY	Subcellular localization
MsCYP79 famliy	*MsCYP1*	MS.gene000461	459	1380	51.83	7.99	-0.29	E R
	MsCYP10	MS.gene008653	493	1482	56.25	8.92	-0.24	E R
	MsCYP107	MS.gene69986	472	1419	53.58	7.99	-0.28	E R
	MsCYP108	MS.gene76445	516	1551	58.43	5.65	-0.29	E R
	MsCYP11	MS.gene008654	459	1380	52.89	9.22	-0.31	E R
	MsCYP110	MS.gene89610	408	1227	46.15	5.03	-0.23	P M
	MsCYP115	MS.gene99572	512	1539	58.03	8.91	-0.32	E R
	MsCYP12	MS.gene008655	494	1485	56.10	8.98	-0.18	E R
	MsCYP16	MS.gene012050	396	1191	45.36	9.20	-0.37	E R
	MsCYP17	MS.gene017540	512	1539	58.04	9.04	-0.33	E R
	MsCYP18	MS.gene017541	472	1419	53.56	8.69	-0.29	E R
	MsCYP2	MS.gene008451	454	1365	51.50	7.20	-0.25	E R
	MsCYP21	MS.gene03927	493	1482	56.35	8.83	-0.26	E R
	MsCYP22	MS.gene03929	466	1401	53.15	8.74	-0.19	E R
	MsCYP23	MS.gene03931	269	810	30.60	8.42	-0.18	E R
	MsCYP24	MS.gene03932	466	1401	53.15	8.74	-0.19	E R
	MsCYP25	MS.gene048532	491	1476	55.85	8.82	-0.20	E R
	MsCYP26	MS.gene048533	271	816	30.74	8.66	-0.17	E R
	MsCYP27	MS.gene048534	492	1479	56.10	8.92	-0.22	E R
	MsCYP28	MS.gene049551	533	1602	60.65	8.53	-0.20	E R
	MsCYP29	MS.gene049552	506	1521	57.70	8.04	-0.25	E R
	MsCYP3	MS.gene008452	454	1365	51.50	7.20	-0.23	E R
	MsCYP30	MS.gene049555	457	1374	51.88	6.26	-0.18	E R
	MsCYP31	MS.gene049560	474	1425	53.85	8.03	-0.28	E R
	MsCYP32	MS.gene049562	474	1425	53.63	8.60	-0.28	E R
	MsCYP33	MS.gene049563	539	1620	61.10	8.88	-0.16	E R
	MsCYP34	MS.gene049576	536	1611	61.30	8.83	-0.21	E R
	MsCYP35	MS.gene05138	518	1557	59.02	8.58	-0.22	E R
	MsCYP36	MS.gene05139	456	1371	51.76	6.65	-0.23	E R
	MsCYP4	MS.gene008464	539	1620	61.18	9.02	-0.18	E R
	MsCYP40	MS.gene05752	494	1485	56.10	9.02	-0.21	E R
	MsCYP41	MS.gene05753	468	1407	53.85	8.49	-0.19	E R
	MsCYP42	MS.gene05756	492	1479	56.11	8.91	-0.25	E R
	MsCYP5	MS.gene008465	437	1314	49.61	9.01	-0.32	E R
	MsCYP53	MS.gene066483	472	1419	53.53	8.57	-0.31	E R
	MsCYP54	MS.gene23793	472	1419	53.58	8.73	-0.30	E R
	MsCYP55	MS.gene23794	512	1539	58.03	8.98	-0.31	E R
	MsCYP56	MS.gene23830	472	1419	53.48	8.57	-0.29	E R
	MsCYP6	MS.gene008466	347	1044	38.94	6.84	-0.22	E R

	MsCYP60	MS.gene27240	512	1539	58.02	9.09	-0.32	E R
	MsCYP61	MS.gene27241	512	1539	58.03	8.98	-0.32	E R
	MsCYP67	MS.gene38420	539	1620	61.21	8.88	-0.15	E R
	MsCYP68	MS.gene38422	527	1584	59.90	9.28	-0.15	E R
	MsCYP69	MS.gene38425	458	1377	52.17	6.16	-0.15	E R
	MsCYP7	MS.gene008467	358	1077	40.91	8.07	-0.05	E R
	MsCYP70	MS.gene38426	507	1524	57.68	8.84	-0.15	E R
	MsCYP71	MS.gene38427	504	1515	57.44	7.69	-0.25	E R
	MsCYP72	MS.gene38428	528	1587	60.22	8.53	-0.22	E R
	MsCYP73	MS.gene38433	505	1518	57.37	7.18	-0.18	E R
	MsCYP74	MS.gene41475	539	1620	61.09	8.86	-0.17	E R
	MsCYP75	MS.gene41476	460	1383	51.92	6.71	-0.23	E R
	MsCYP76	MS.gene41477	425	1278	47.52	8.15	-0.16	E R
	MsCYP77	MS.gene41481	457	1374	52.01	6.07	-0.16	E R
	MsCYP78	MS.gene41482	524	1575	59.66	8.95	-0.19	E R
	MsCYP79	MS.gene41484	457	1374	51.90	6.11	-0.19	E R
	MsCYP8	MS.gene008470	457	1374	51.85	6.07	-0.18	E R
	MsCYP80	MS.gene41487	505	1518	57.63	8.48	-0.25	E R
	MsCYP81	MS.gene41488	533	1602	60.74	8.53	-0.20	E R
	MsCYP9	MS.gene008474	454	1365	51.67	7.98	-0.29	E R
	MsCYP100	MS.gene50229	421	1266	48.79	6.00	-0.21	P M
	MsCYP101	MS.gene63983	515	1548	59.13	8.08	-0.17	P M
	MsCYP102	MS.gene63984	335	1008	38.61	5.03	-0.21	P M
	MsCYP103	MS.gene64188	430	1293	49.55	6.44	-0.25	P M
	MsCYP104	MS.gene64854	221	666	25.35	6.00	-0.03	P M
	MsCYP105	MS.gene67549	288	867	33.27	5.45	-0.09	P M
	MsCYP106	MS.gene67575	345	1038	39.68	5.47	-0.08	P M
	MsCYP109	MS.gene76640	345	1038	39.32	5.33	-0.11	P M
	MsCYP111	MS.gene97180	384	1155	43.72	6.15	-0.24	P M
	MsCYP112	MS.gene97182	275	828	31.41	5.51	-0.41	E R
	MsCYP113	MS.gene97561	324	975	36.95	5.86	-0.33	P M
MsCYP83	*MsCYP114*	MS.gene97562	384	1155	43.72	6.15	-0.24	P M
famliy	*MsCYP13*	MS.gene009779	350	1053	40.16	5.28	-0.15	P M
	MsCYP14	MS.gene009780	362	1089	41.64	5.54	-0.25	P M
	MsCYP15	MS.gene009782	362	1089	41.61	5.67	-0.25	P M
	MsCYP19	MS.gene021849	299	900	34.12	5.23	-0.31	E R
	MsCYP20	MS.gene021850	322	969	36.55	5.74	-0.32	P M
	MsCYP37	MS.gene057498	324	975	36.99	5.74	-0.28	P M
	MsCYP38	MS.gene057499	365	1098	41.66	6.39	-0.37	P M
	MsCYP39	MS.gene057506	385	1158	43.99	6.43	-0.23	P M
	MsCYP43	MS.gene059229	341	1026	39.62	5.51	-0.34	P M
	MsCYP44	MS.gene059230	345	1038	39.50	5.60	-0.15	P M
	MsCYP45	MS.gene059234	336	1011	39.06	5.50	-0.21	P M
	MsCYP46	MS.gene059235	344	1035	39.48	6.32	-0.08	P M

MsCYP47	MS.gene059236	345	1038	39.20	5.46	-0.11	P M
MsCYP48	MS.gene059237	275	828	31.67	5.67	-0.31	E R
MsCYP49	MS.gene059238	275	828	31.74	5.10	-0.24	E R
MsCYP50	MS.gene059239	328	987	37.82	5.15	-0.19	P M
MsCYP51	MS.gene059241	201	606	22.64	5.44	-0.09	E R
MsCYP52	MS.gene059243	345	1038	39.32	5.33	-0.11	P M
MsCYP57	MS.gene24192	431	1296	49.83	6.19	-0.27	P M
MsCYP58	MS.gene24193	515	1548	59.00	8.56	-0.17	P M
MsCYP59	MS.gene27037	430	1293	49.65	6.30	-0.26	P M
MsCYP62	MS.gene35889	421	1266	48.76	6.00	-0.19	P M
MsCYP63	MS.gene35893	362	1089	41.61	5.54	-0.24	P M
MsCYP64	MS.gene35895	506	1521	57.94	8.62	-0.20	P M
MsCYP65	MS.gene35896	478	1437	54.79	8.84	-0.24	P M
MsCYP66	MS.gene35897	362	1089	41.57	5.67	-0.25	P M
MsCYP82	MS.gene45860	255	768	29.35	5.09	-0.18	E R
MsCYP83	MS.gene45866	202	609	23.11	6.22	-0.16	E R
MsCYP84	MS.gene45867	317	954	36.65	6.28	-0.09	P M
MsCYP85	MS.gene45868	345	1038	39.36	5.14	-0.10	P M
MsCYP86	MS.gene45870	344	1035	39.46	5.48	-0.19	P M
MsCYP87	MS.gene45871	328	987	37.85	5.22	-0.19	P M
MsCYP88	MS.gene45873	497	1494	56.78	6.84	-0.03	P M
MsCYP89	MS.gene48260	292	879	33.96	5.32	-0.29	P M
MsCYP90	MS.gene48262	201	606	22.87	6.84	-0.14	E R
MsCYP91	MS.gene48269	393	1182	45.46	6.37	-0.19	P M
MsCYP92	MS.gene48905	328	987	37.74	5.13	-0.17	P M
MsCYP93	MS.gene48906	275	828	31.72	5.02	-0.22	E R
MsCYP94	MS.gene48907	275	828	31.72	5.55	-0.32	E R
MsCYP95	MS.gene48911	344	1035	39.67	6.08	-0.09	P M
MsCYP96	MS.gene48913	345	1038	39.47	5.41	-0.11	P M
MsCYP97	MS.gene49206	421	1266	48.62	6.40	-0.26	P M
MsCYP98	MS.gene49208	362	1089	41.63	5.54	-0.25	P M
MsCYP99	MS.gene50228	421	1266	48.66	6.17	-0.27	P M

Note: GRAVY: Grand average of hydropathicity; E R: Endoplasmic Reticulum;P M: Plasma Membrane.
