# Supplementary material for: Identification of MsCYP79 and MsCYP83 gene families and its response to mechanical damage in Medicago sativa L
Source: PLoS One. 2025 May 8;20(5):e0322981. doi: 10.1371/journal.pone.0322981 (PMC12061124; doi:10.1371/journal.pone.0322981)
Supplement: S2 Table — (PDF) [file pone.0322981.s002.pdf]

S2 Table cis-acting elements in the promoter region of MsCYP79 and MsCYP83 family genes

	Gene name	CAAT -box	TATA-box	MYB	MYC	ABRE	ARE	Box 4	G-box	GT1-motif	AT-TATA-box	ERE	STRE
MsCYP79 famliy	*MsCYP1*	35	26	5	6	1	1	2	0	5	3	0	2
	MsCYP10	35	18	5	4	1	2	2	1	4	0	2	0
	MsCYP107	42	21	7	6	0	2	5	1	2	2	0	3
	MsCYP108	52	18	5	6	5	3	1	5	0	3	0	7
	MsCYP11	39	30	1	4	3	3	8	3	0	6	0	3
	MsCYP110	39	17	4	1	5	2	1	5	3	4	1	2
	MsCYP115	46	22	6	5	0	0	6	1	2	2	0	3
	MsCYP12	59	15	4	5	1	3	1	3	0	3	0	1
	MsCYP16	41	28	7	3	3	0	1	3	1	5	0	2
	MsCYP17	44	35	4	7	0	1	6	0	2	5	2	1
	MsCYP18	39	24	4	6	1	1	1	0	4	3	0	3
	MsCYP2	30	21	3	5	2	1	3	2	1	0	5	2
	MsCYP21	35	18	5	4	1	2	2	1	4	1	0	2
	MsCYP22	34	29	6	6	1	4	1	2	2	1	1	0
	MsCYP23	43	29	2	4	2	5	3	2	0	7	0	1
	MsCYP24	34	29	6	6	1	4	1	2	2	1	1	0
	MsCYP25	36	22	4	6	2	4	2	3	1	3	0	4
	MsCYP26	39	31	1	4	2	5	2	2	0	8	0	2
	MsCYP27	29	14	6	4	1	2	1	1	4	1	0	4
	MsCYP28	43	37	6	5	3	2	8	3	2	5	2	0
	MsCYP29	16	38	3	3	1	1	1	2	1	5	2	7
	MsCYP3	38	21	3	5	2	2	1	3	0	0	3	5
	MsCYP30	28	30	3	2	1	2	2	2	2	3	1	1
	MsCYP31	37	19	2	0	8	3	4	8	1	0	1	2
	MsCYP32	23	34	2	3	0	4	1	0	1	6	3	2
	MsCYP33	26	25	2	2	1	2	3	3	0	1	1	4
	MsCYP34	37	18	5	4	2	2	4	2	0	2	5	1
	MsCYP35	37	17	4	5	1	2	4	1	0	2	5	1
	MsCYP36	41	23	4	5	2	2	1	3	0	0	3	5
	MsCYP4	24	27	2	3	1	2	3	3	0	1	1	1
	MsCYP40	41	24	4	7	2	4	4	3	2	3	0	3
	MsCYP41	33	23	1	1	5	6	2	5	0	7	1	3
	MsCYP42	36	18	3	5	0	1	1	0	3	1	1	5
	MsCYP5	24	34	2	3	0	4	1	0	1	5	3	2
	MsCYP53	41	21	6	6	1	1	5	2	2	2	0	2
	MsCYP54	20	11	0	4	3	4	2	5	1	2	2	4
	MsCYP55	44	27	8	10	1	2	4	1	1	3	1	2
	MsCYP56	40	19	7	6	0	2	5	1	2	2	0	3
	MsCYP6	45	15	3	7	0	5	3	1	0	1	0	1

	MsCYP60	47	37	2	6	0	5	7	0	1	6	3	1
	MsCYP61	41	26	3	4	0	0	2	0	3	3	0	2
	MsCYP67	47	18	6	5	3	0	4	4	4	0	1	1
	MsCYP68	28	30	4	3	0	19	0	1	0	3	1	1
	MsCYP69	30	34	0	4	0	2	3	0	2	3	4	3
	MsCYP7	36	15	8	5	1	1	0	1	1	2	1	2
	MsCYP70	46	18	5	0	0	2	3	0	2	1	1	5
	MsCYP71	18	43	2	4	2	1	2	1	0	6	3	6
	MsCYP72	43	35	5	5	3	2	7	3	1	5	2	1
	MsCYP73	41	34	7	2	3	1	6	3	3	3	1	3
	MsCYP74	27	26	1	3	2	2	3	2	0	1	1	2
	MsCYP75	44	19	3	7	1	6	3	2	1	2	0	0
	MsCYP76	10	17	1	1	5	1	6	8	2	1	4	5
	MsCYP77	31	34	0	4	1	2	3	1	2	3	5	3
	MsCYP78	43	19	5	0	0	2	3	0	2	1	0	5
	MsCYP79	24	31	0	3	1	2	4	1	1	3	2	4
	MsCYP8	27	32	3	1	2	2	3	3	2	3	1	1
	MsCYP80	30	34	3	4	2	1	1	1	0	4	6	5
	MsCYP81	39	33	5	1	2	1	6	1	2	4	1	3
	MsCYP9	35	35	6	5	1	3	6	1	1	3	3	0
	MsCYP100	30	30	2	8	4	2	2	3	1	0	0	0
	MsCYP101	57	20	2	4	2	4	0	2	1	0	4	0
	MsCYP102	39	21	5	3	1	8	2	1	0	1	2	1
	MsCYP103	37	24	3	1	1	2	0	1	0	0	6	2
	MsCYP104	51	24	2	8	2	2	6	2	2	3	1	4
	MsCYP105	37	32	4	3	1	3	3	1	0	5	0	3
	MsCYP106	46	23	6	4	2	2	6	2	2	3	1	3
	MsCYP109	32	17	5	3	2	3	0	5	1	1	2	3
	MsCYP111	46	23	4	7	0	0	3	1	0	1	4	6
	MsCYP112	35	14	6	6	0	3	3	0	1	3	1	2
	MsCYP113	39	27	5	9	1	2	1	1	3	2	2	1
MsCYP83	*MsCYP114*	43	21	2	9	2	1	1	3	0	0	3	2
famliy	*MsCYP13*	41	32	4	3	1	2	3	2	1	1	1	6
	MsCYP14	40	17	1	4	0	6	0	0	0	2	1	1
	MsCYP15	26	30	2	6	0	6	3	0	1	1	5	6
	MsCYP19	50	35	2	11	1	2	1	1	0	1	2	1
	MsCYP20	47	28	1	6	0	4	3	1	3	2	2	1
	MsCYP37	51	20	6	10	1	7	0	1	1	0	0	2
	MsCYP38	31	30	1	4	3	1	3	1	0	3	3	1
	MsCYP39	31	29	1	4	1	1	2	0	0	3	4	1
	MsCYP43	21	30	1	2	5	3	2	6	5	1	11	5
	MsCYP44	39	27	7	6	2	4	3	2	1	1	0	1
	MsCYP45	36	18	3	6	1	2	1	3	0	0	4	6
	MsCYP46	34	38	1	1	1	1	0	2	0	3	1	1

MsCYP47	53	20	2	8	2	4	2	2	1	1	0	0
MsCYP48	36	23	6	3	3	3	1	3	1	3	0	0
MsCYP49	32	27	5	2	3	3	1	3	0	5	1	0
MsCYP50	36	31	5	3	2	3	0	1	0	6	0	0
MsCYP51	44	20	2	6	1	5	2	1	2	2	1	1
MsCYP52	32	16	6	3	2	3	0	5	1	1	2	3
MsCYP57	54	33	3	3	3	5	2	2	0	4	4	0
MsCYP58	48	33	4	4	3	1	0	3	1	1	8	0
MsCYP59	50	26	4	3	1	6	4	1	1	3	2	0
MsCYP62	31	30	2	9	4	2	2	3	1	1	0	1
MsCYP63	41	15	1	4	2	5	2	2	1	3	0	1
MsCYP64	43	20	4	5	0	5	2	0	1	1	1	1
MsCYP65	38	21	1	2	2	8	1	2	1	5	5	0
MsCYP66	26	25	2	4	0	7	1	0	0	2	3	1
MsCYP82	38	23	8	7	3	2	4	3	3	1	0	0
MsCYP83	26	42	1	1	2	1	4	3	1	7	0	11
MsCYP84	37	41	1	2	1	1	0	3	0	4	1	0
MsCYP85	44	35	1	6	3	4	1	3	1	5	1	0
MsCYP86	46	23	6	6	1	3	4	2	0	2	1	1
MsCYP87	35	44	7	2	1	1	1	2	2	7	1	0
MsCYP88	35	27	3	2	3	5	2	2	2	3	0	0
MsCYP89	29	32	2	1	5	2	1	7	3	1	7	6
MsCYP90	41	35	1	5	1	2	3	2	0	6	1	1
MsCYP91	41	47	1	7	4	3	1	4	2	4	1	0
MsCYP92	35	23	7	2	3	5	0	2	0	5	0	0
MsCYP93	31	24	5	2	3	3	1	3	0	4	1	0
MsCYP94	34	25	6	3	3	3	1	3	1	4	0	0
MsCYP95	33	45	1	2	4	3	1	4	3	5	4	1
MsCYP96	45	52	2	6	2	4	3	2	1	5	1	1
MsCYP97	28	28	2	6	0	6	3	0	1	1	5	6
MsCYP98	38	17	1	3	0	6	0	0	0	2	1	1
MsCYP99	33	15	2	4	1	5	2	1	2	3	0	2
