# Supplementary material for: Identification of MsCYP79 and MsCYP83 gene families and its response to mechanical damage in Medicago sativa L
Source: PLoS One. 2025 May 8;20(5):e0322981. doi: 10.1371/journal.pone.0322981 (PMC12061124; doi:10.1371/journal.pone.0322981)
Supplement: S3 Table — (PDF) [file pone.0322981.s003.pdf]

S3 Table Primers used for RT-qPCR analysis

Gene name	Primer sequence (5’ - 3’)
18S rRNA	F: CTGAGAAACGGCTACCACATC<br>R: CAACCCAAGGTCCAACTACGAG
MsCYP43	F: CAAAGATTATCTTATAATGGAAGGT<br>R: GCACGAAGGCCAGATAGTTTAT
MsCYP89	F: AGGATGAAGGAACCAAGAGGAG<br>R:CCCAAACTATTGTGGCTGACG
MsCYP71	F:TCATCTCCAGTGGATACTTGACCTC<br>R:ATCGGCTTCTTCGTTCCTTT
MsCYP102	F:TATTTGTGATTATGAAGAAGAAGTT<br>R:AAAAGTAAAATTCGGTCAACAAAGC
MsCYP50	F:GCACCTACCAGCCCCTCTACTT<br>R:AAGGATAAGTTCCAATGAGGCA
