# Supplementary figures and images for: Identification of MsCYP79 and MsCYP83 gene families and its response to mechanical damage in Medicago sativa L
Source: PLoS One. 2025 May 8;20(5):e0322981. doi: 10.1371/journal.pone.0322981 (PMC12061124; doi:10.1371/journal.pone.0322981)

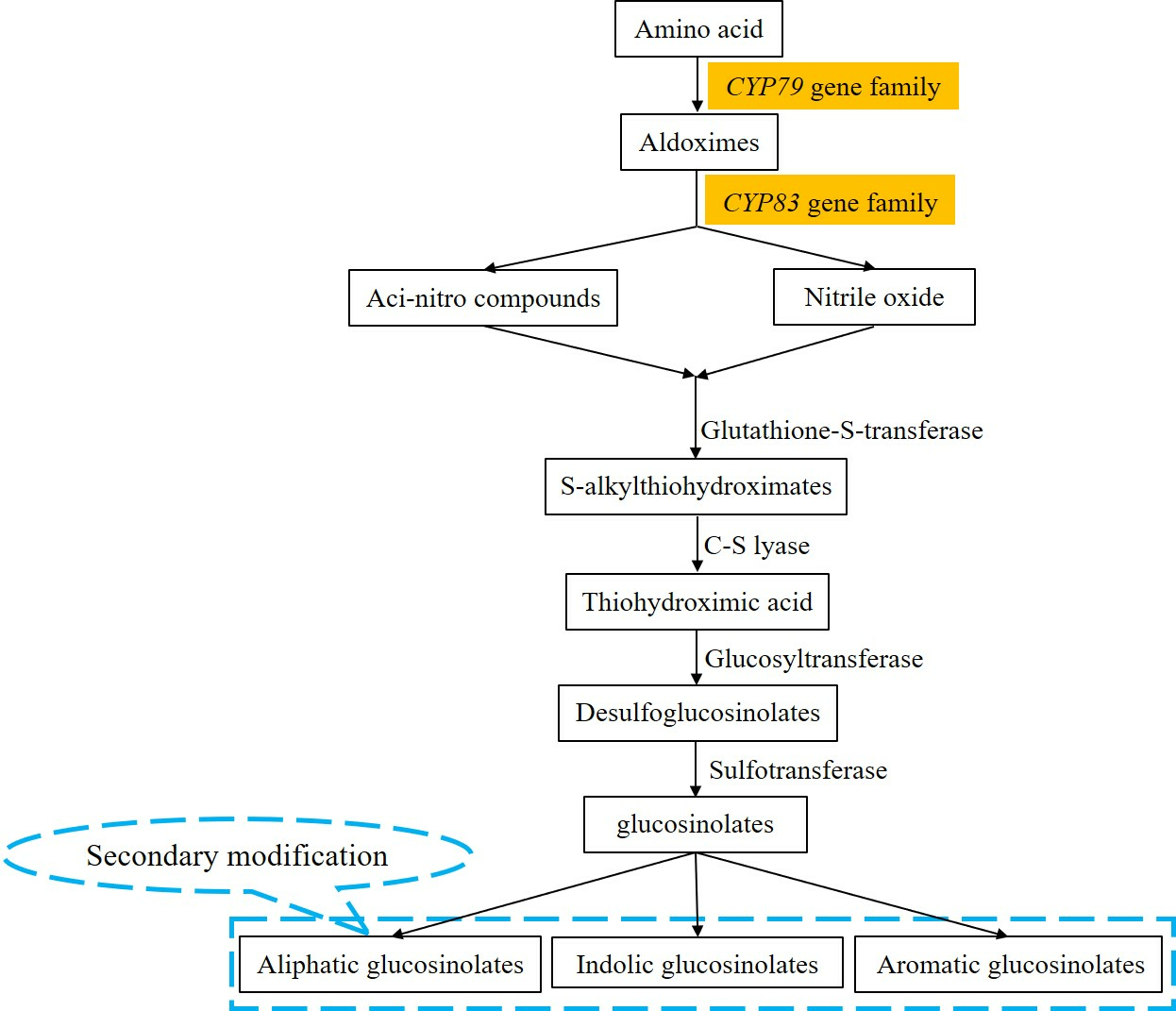

Supplement: S1 Fig — (TIF) [file pone.0322981.s004.tif]

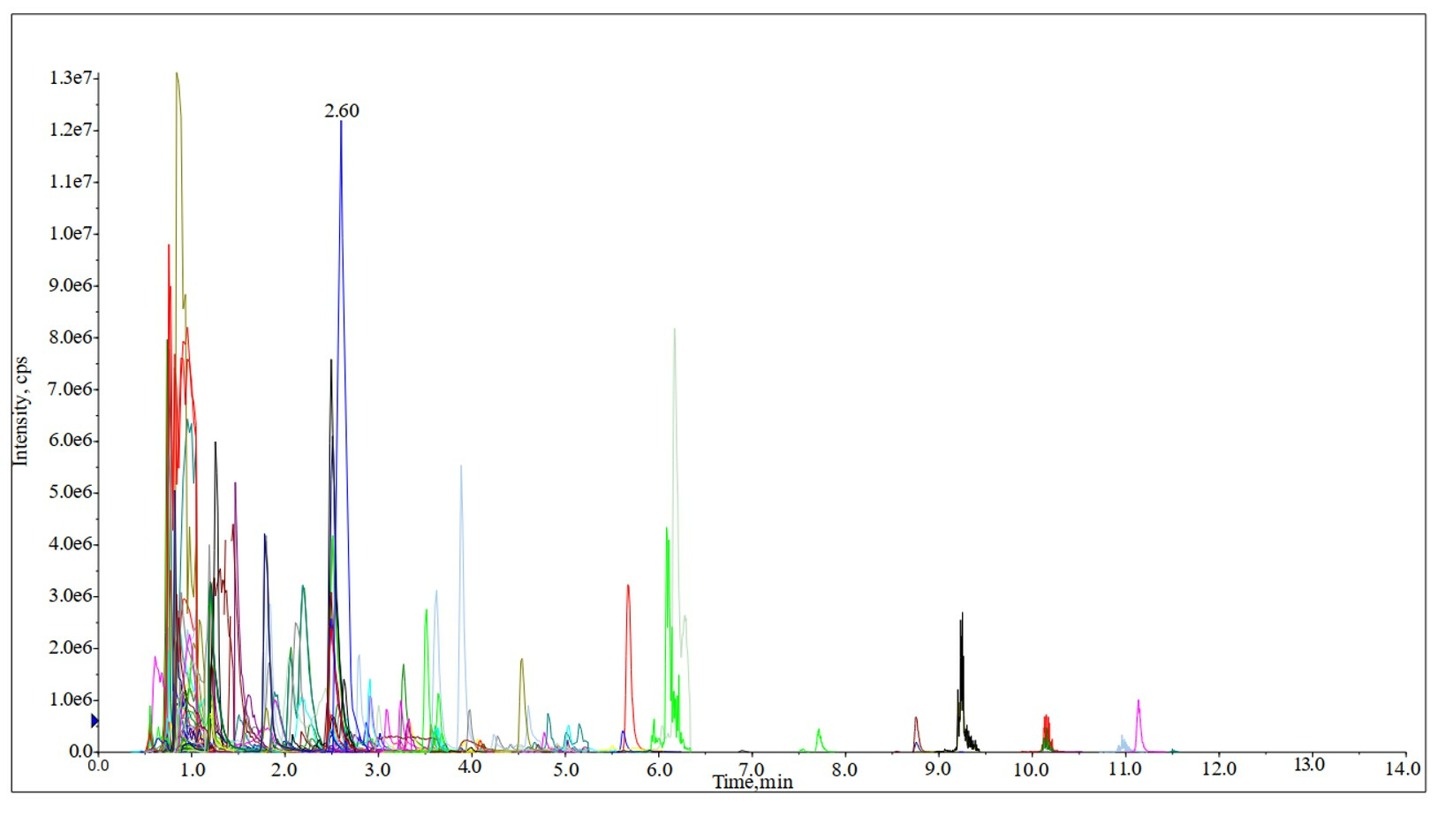

Supplement: S2 Fig — (TIF) [file pone.0322981.s005.tif]

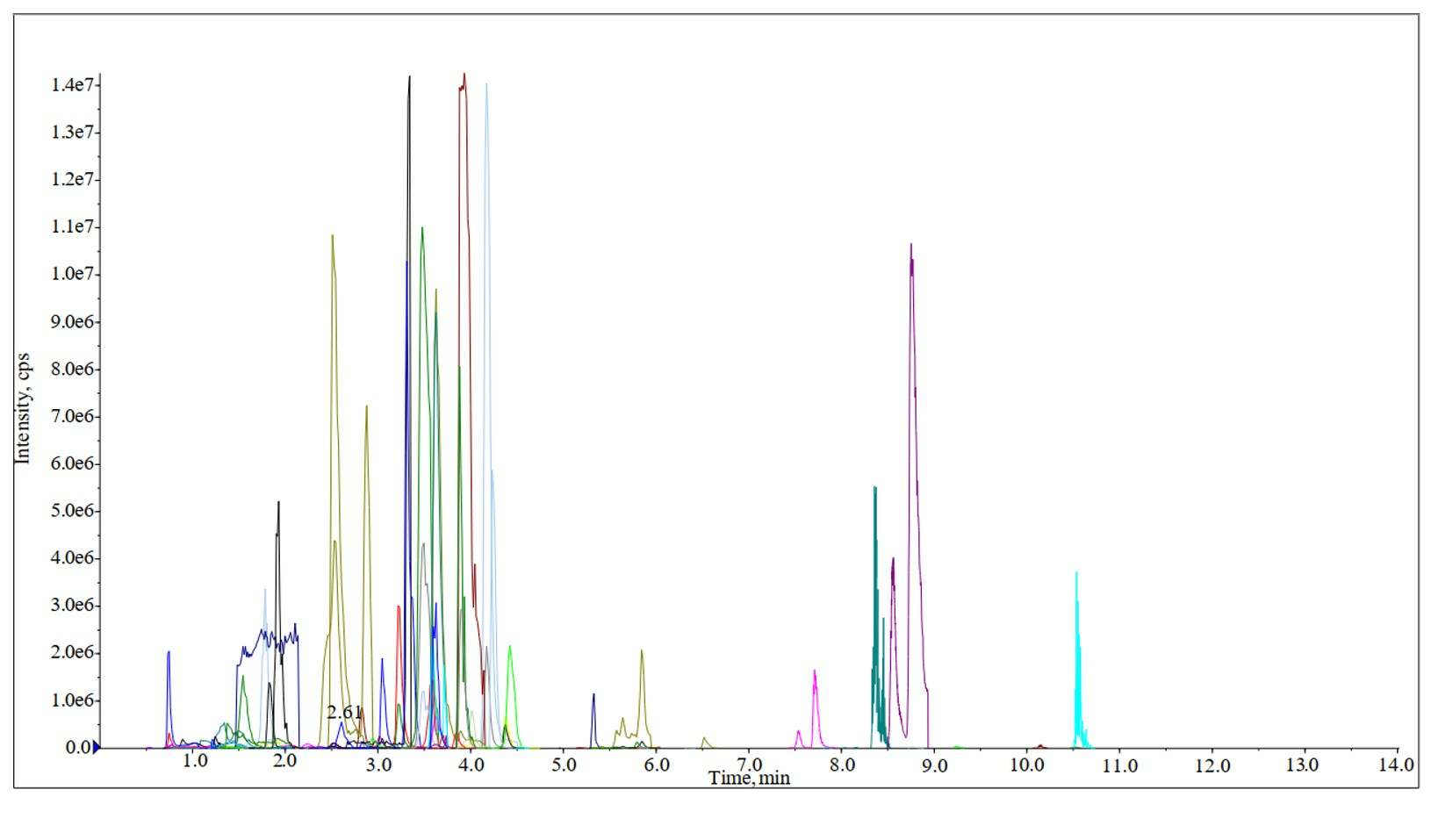

Supplement: S3 Fig — (TIF) [file pone.0322981.s006.tif]

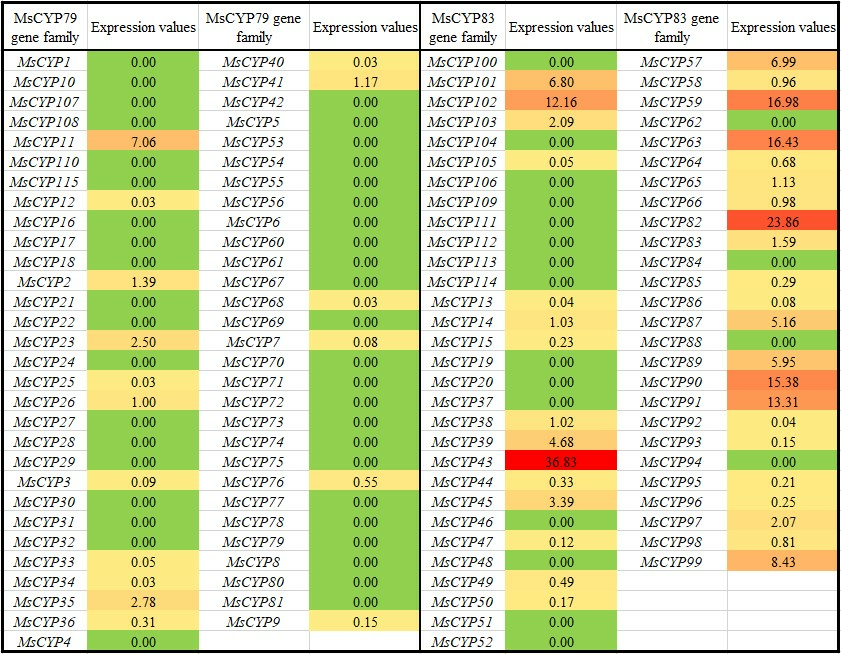

Supplement: S4 Fig — Gannong NO.9. (TIF) [file pone.0322981.s007.tif]
